# Glutamine-to-glutamate ratio in the nucleus accumbens predicts effort-based motivated performance in humans

**DOI:** 10.1038/s41386-020-0760-6

**Published:** 2020-07-20

**Authors:** Alina Strasser, Gediminas Luksys, Lijing Xin, Mathias Pessiglione, Rolf Gruetter, Carmen Sandi

**Affiliations:** 1 Laboratory of Behavioral Genetics (LGC), Brain Mind Institute (BMI), Ecole Polytechnique Fédérale de Lausanne (EPFL), Lausanne, Switzerland; 2 Centre for Discovery Brain Sciences (CDBS), University of Edinburgh, Edinburgh, UK; 3 ZJU-UoE Institute, Zhejiang University International Campus, Haining, China; 4 Animal Imaging and Technology Core (AIT), Center for Biomedical Imaging (CIBM), Ecole Polytechnique Fédérale de Lausanne (EPFL), Lausanne, Switzerland; 5 Motivation, Brain and Behavior Team, Brain and Spine Institute (ICM), Paris, France; 6 Laboratory of Functional and Metabolic Imaging, Ecole Polytechnique Fédérale de Lausanne (EPFL), Lausanne, Switzerland; 7 Department of Radiology, University of Lausanne (UNIL), Lausanne, Switzerland; 8 Department of Radiology, University of Geneva (UNIGE), Geneva, Switzerland

**Keywords:** Motivation, Stress and resilience

## Abstract

Substantial evidence implicates the nucleus accumbens in motivated performance, but very little is known about the neurochemical underpinnings of individual differences in motivation. Here, we applied ^1^H magnetic resonance spectroscopy (^1^H-MRS) at ultra-high-field in the nucleus accumbens and inquired whether levels of glutamate (Glu), glutamine (Gln), GABA or their ratios predict interindividual differences in effort-based motivated task performance. Given the incentive value of social competition, we also examined differences in performance under self-motivated or competition settings. Our results indicate that higher accumbal Gln-to-Glu ratio predicts better overall performance and reduced effort perception. As performance is the outcome of multiple cognitive, motor and physiological processes, we applied computational modeling to estimate best-fitting individual parameters related to specific processes modeled with utility, effort and performance functions. This model-based analysis revealed that accumbal Gln-to-Glu ratio specifically relates to stamina; i.e., the capacity to maintain performance over long periods. It also indicated that competition boosts performance from task onset, particularly for low Gln-to-Glu individuals. In conclusion, our findings provide novel insights implicating accumbal Gln and Glu balance on the prediction of specific computational components of motivated performance. This approach and findings can help developing therapeutic strategies based on targeting metabolism to ameliorate deficits in effort engagement.

## Introduction

There are substantial individual differences in human achievement in various life domains, from education to work or sports [1, 2]. Motivation is key to success and an important factor for goal-directed behavior and well-being [3, 4]. Motivational deficits, such as apathy, are prevalent in neurodegeneration and psychiatric disorders [5–7]. Understanding the neurobiological underpinnings that lead to individual differences in motivated performance can help developing new strategies to ameliorate deficits in reward valuation and effort engagement.

In agreement with the rodent literature [8, 9], the ventral striatum, including the nucleus accumbens, has emerged in humans as a key component of the motivation brain circuitry regulating motivated behavior [10–14]. In addition to reward processing, the nucleus accumbens has been implicated in cost-based behavioral allocation for both mental and physical effort [13]. However, knowledge about neurochemical mechanisms linking accumbal function with motivated behavior is scarce.

Recently, the quantification of metabolites related to excitatory [i.e., glutamate (Glu)] and inhibitory (i.e., GABA) neurotransmission with ^1^H magnetic resonance spectroscopy (^1^H-MRS) has been applied to predict individual differences in memory and decision-making tasks [15–18]. Here, we targeted the nucleus accumbens with ^1^H-MRS at ultra-high-field (7 T), allowing a precise quantification of Glu and GABA, and the effective separation of Glu and its metabolic precursor glutamine (Gln) [19] to predict effort-based motivated performance. Importantly, motivated performance is a complex process that involves multiple behavioral functions [1, 8]. Our aim was to investigate whether resting levels of Glu, Gln, and GABA, and Gln/Glu and GABA/Gln ratios predict specific components of effort-based motivated performance. To provide evidence for the accumbal specificity of these neurochemical–behavioral associations, we also performed ^1^H-MRS in the occipital lobe. Importantly, in addition to their role in neurotransmission, these three metabolites participate in multiple metabolic pathways, including energy production and the synthesis of the antioxidant glutathione [20].

To dissect motivation into its component elements, we used a recently developed effort-based monetary incentivized task [21] that combines aspects from the MID task [10] and effort-based decision-making paradigms [11, 22]. Importantly, this task probes different aspects of motivated performance while capturing a wide range of individual differences [21]. In addition, in order to account for the modulation of performance by situational factors [23, 24], and given the capacity of social competition to improve performance in a variety of settings [25–28], we compared performance under competition versus self-motivated performance.

Then, we applied computational modeling designed to dissect performance in this task to specific components such as curvature of the utility function, sprint and endurance stamina, performance baseline and its randomness. Previous studies [29–32] have shown that computational approaches enhance our insight to behavioral mechanisms and reveal otherwise inaccessible neurobiological correlates. Here, applying computational modeling allowed us to reveal critical associations between metabolites, or their ratios, and particular components underlying motivated performance (e.g., utility curvature, stamina, and other performance parameters). We show that the accumbal ratio of glutamine-to-glutamate specifically predicts effortful performance, by particularly relating to the stamina required to keep up performance throughout the task.

## Materials and methods

For complete information on Materials and Methods, please see Supplementary Methods

### Participants

From 43 men, 20–30 years old, originally recruited for the study, we obtained valid MRS data from 27 of them (i.e., data from 16 participants could not be fully collected and included in the analysis; see specific reasons in Participants section in Supplementary Methods). Participants were characterized for several personality measurements (see section on Personality questionnaires and anthropometric characteristics in Supplementary Methods). Data analyzed here is part of a larger study from which a report on the link of metabolites with anxiety trait has been previously published [33]. Informed consent was obtained from all participants in the study. Experiments were performed in accordance with the Declaration of Helsinki and approved by the Cantonal Ethics Committee of Vaud, Switzerland. See Supplementary Methods for further details.

### Proton magnetic resonance spectroscopy (^1^H MRS) acquisition and data processing

**Fig. 1:**
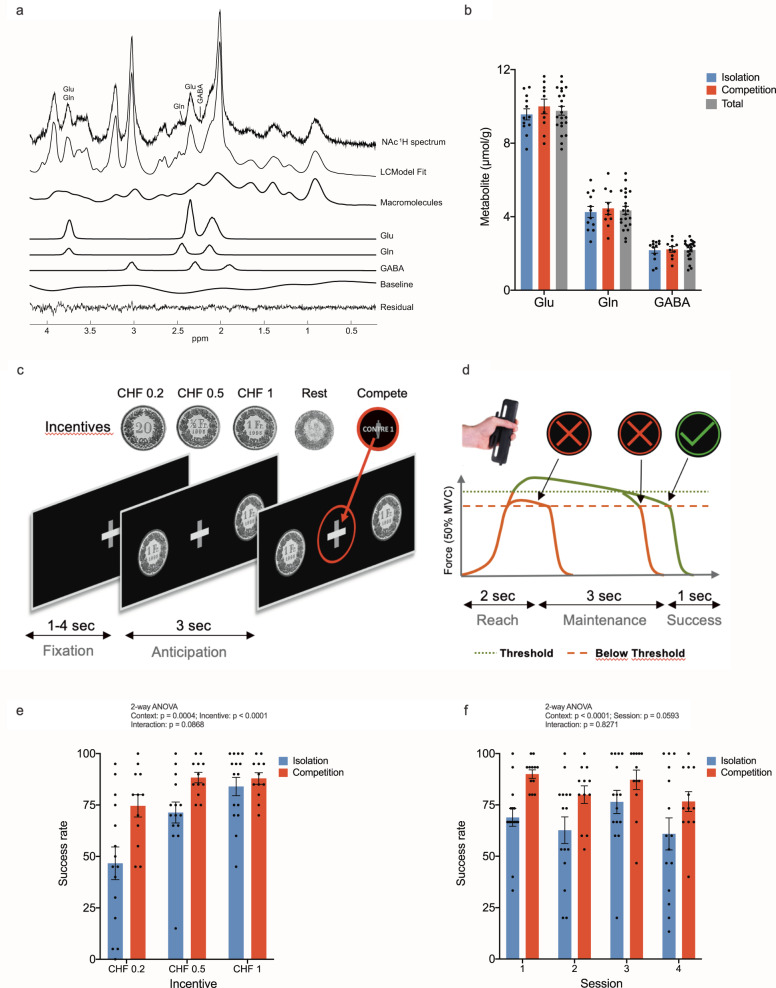
In vivo ^1^H MR spectroscopy in the nucleus accumbens at rest and experimental design of behavioral testing. **a** This panel includes a representative ^1^H MR spectrum acquired with the semi-adiabatic SPECIAL sequence at 7 T (TE/TR = 16 ms/6500 ms, 256 averages), as well as the corresponding LCModel spectral fit, fit residual, macromolecules, baseline and individual metabolite fits for glutamate (Glu), glutamine (Gln), and GABA. **b** Accumbal metabolite concentrations for glutamate, glutamine, and GABA. No differences in metabolite concentrations were observed between the experimental groups (two-sided independent Student’s *t* test), indicating optimal group matching for these metabolite concentrations. No differences in metabolite concentrations were expected between the experimental groups (i.e., between isolation and competition) at baseline, as MRS acquisition took place before the social manipulation. Mean metabolite concentrations for both groups combined are also shown (marked Total in the gray bar). Glu glutamate, Gln glutamine, GABA gamma-aminobutyric acid. Error bars are shown in standard deviations. **c** Modified incentive delay task and the performance of the different participants in the isolation and competition context. Visual stimuli of the modified monetary incentive delay task in the CHF 1 isolation condition. In the competition condition, the gray plus sign was replaced with a gray cartoon of a male opponent, overlaid by the text “against 1”. Successive screen images were shown to participants whilst they were performing the hand grip task, and which guided and cued their performance in line with the instructions that were received prior to data acquisition. We ensured that all participants had seen all visual stimuli and understood the task during the 20-trial experimental practice session. **d** Exemplary trial dynamic. Here, we show the force dynamics that would occur after the 3 s anticipation period shown in (**c**). The anticipation period was always followed by a 2 s period during which the force threshold of 50% of their maximal voluntary contraction (MVC) had to be reached. Then, participants had to maintain the force at their threshold level for another 3 s. If successful in this, a green tick appeared on the screen for 1 s, if not, a red cross. **e** This plot shows the success rate of each participant (indicated as a dot), in the isolation and the competition condition. Performance is shown as a function of the three different incentive sizes (CHF 0.2, CHF 0.5, CHF 1) and of the isolation and competition contexts. In **f** participants’ performance is plotted as a function of session number (1–4) and of the isolation and competition contexts. The task was structured into two blocks, separated by a 3 min break. Each block contained 2 sessions of each 20 trials: 5 rest trials that occurred at an interval of every 3 action trials, with the incentive sizes varying pseudorandomly to ensure that each incentive could be earned 5 times. The entire MIF task comprised 80 trials. Error bars, SEM. *n* = 15; isolation. *n* = 12, competition.

The MR measurements were performed on a Magnetom 7 T/68-cm head scanner (Siemens, Erlangen, Germany) equipped with a single-channel quadrature transmit and a 32-channel receive coil (Nova Medical Inc., MA, USA). The NAc region of interest voxel (VOI) was defined by the third ventricle medially, the subcallosal area inferiorly, and the body of the caudate nucleus and the putamen laterally and superiorly, in line with definitions of NAc anatomy identifiable on MRIs [34] (Supplementary Fig. 1). A representative spectrum of the NAc voxel is shown in Fig. 1a. We obtained an overall spectral SNR and linewidth of 72 ± 9 and 0.048 ± 0.006 ppm, respectively. Glu, Gln, and GABA concentrations in the NAc were quantified with CRLB of 2.36 ± 0.49%, 4.91 ± 0.75% and 11.32 ± 2.78% (Supplementary Table 2). Given that, in order to obtain high-quality MRS measurements in the NAc in the 7 T requires 90 min of scanning time, MRS acquisition in a second, control brain region had to be postponed to a subsequent session. Thus, we obtained spectra from the occipital lobe as an experimental control on 17 participants that were successfully recruited for a second scanner. See Supplementary Methods for further details.

### Effort-related monetary incentive force task

Our modified monetary incentive delay (MID) task [10] version [21] relied on exerting force on a hand grip or dynamometer (TSD121B-MRI, Biopac) (Fig. 1d) at a threshold corresponding to 50% of each participant’s maximum voluntary contraction (MVC) and, therefore, was termed monetary incentive force (MIF) task. To investigate the influence of competition on performance, the experiment was run under two experimental conditions, an isolation and a competition condition. Success rate was computed in % of successful trials out of total trials, and for each of the four sessions (i.e., Success_Total_, Success_Session 1_, Success_Session 2_, Success_Session 3_, and Success_Session 4_) and for each of the three incentives (i.e., CHF 0.2, 0.5, and 1). See Supplementary Methods for further details.

### Computational modeling

To study more intricate aspects of motivated performance, we developed a computational model thereof. As described above, participants had to reach the same threshold for successful performance with different incentives (CHF 0.2, 0.5, and 1), and their performance was energetically costly since force had to be exerted. Hence, at each trial, subjects were primed with different monetary incentive values and they subsequently had to decide whether to invest energy in this trial and to perform the task accordingly. To build our model, we consequently assumed that (i) metabolic resources gradually decline during the task along with the cumulative effort exerted by participants and that (ii) a certain degree of metabolic recovery will take place during the 3 min resting break that participants were given between the two task blocks. We termed these two components participants’ sprint stamina (*ε*_spr_) and endurance stamina (*ε*_end_): *ε*_spr_ quantified the fraction of initial energy remaining at the beginning of session 2 of each of the two blocks and *ε*_end_ quantified the fraction of initial energy remaining at the beginning of session 3 (i.e., after the 3 min break) to account for recovery experienced during the break.

Subjective utility (i.e., how nominal value relates to perceived value) was modeled using a difference of a common power law function [35] for reward gains and effort costs [36] as

1 $$u\left( x \right) = x^\alpha - \tau ,$$

where *x*^*α*^ is the reward gain component and τ the effort cost component. For the reward gain component, *x* is monetary value (CHF 0.2, 0.5, and 1) and parameter *α* (utility curvature) describes how much participants valued high versus low incentives. Values of *α* under 1 reflected concave utility, which is very common in behavioral economics. For the effort cost component, participants’ average energy level during a session was modeled using function *E* with *E* = 1 during session 1 (referring to the initial amount of energy) and *E* = ε_spr_, *E* = *ε*_end_, and *E* = *ε*_spr_*ε_end_ during sessions 2–4, respectively (with 0 < *ε*_spr_ < 1 and 0 < *ε*_end_ < 1, as energy was expended during continuous performance but partially recovered during the break). Then the effort component *τ* consisted of effort cost baseline *b* and energy loss 1 − *E*:

2 $$\tau = {b} + 2(1 - E).$$

Consequently, *τ* was equal to *b* when the participant had full energy and *b* + 1 when *E* = 0.5. It might seem appropriate to have included another parameter *b*_max_ instead of 2 (a constant) to define an upper limit to *τ*, but as monetary values could also be adjusted arbitrarily on the gain side (e.g., 20/50/100 instead of 0.2/0.5/1) to counter its effect, having arbitrary scaling of *τ* (and an extra free parameter) was unnecessary, as free parameters of the effort cost function (*b*, *ε*_spr_, and *ε*_end_) provided sufficient flexibility.

We modeled probability of success in a trial (reaching the necessary force threshold and maintaining it for 3 s) using a sigmoidal function of subjective utility *u*(*x*) and sigmoidal steepness (inverse temperature) *β*, which determined choice randomness (higher *β* = less randomness)

3 $$P(\rm{success}) = \frac{1}{{1 + e^{ - u\beta }}}.$$

Our model is similar to Le Bouc et al. [36], with the main differences being that our model uses utility curvature for rewards (common in behavioral economics), our effort cost baseline b is related to effort sensitivity divided by reward sensitivity in Le Bouc et al. [36], and 1 − *E* (energy loss) to susceptibility to fatigue/fatiguability there. We modeled loss of energy exponentially as opposed to linearly, as it may be a better approximation. Because of the nature of our data, we also used discrete points (averages of four sessions) instead of continuous time. Hence, our stamina parameters are closely linked to fatiguability parameter *K*_*f*_ in Le Bouc et al. [36].

Twelve performance measures (PMs) were chosen: success rates in each of the 4 sessions and for each of the 3 incentives. To evaluate how well the model fits participants’ performance, a goodness of fit function was used [29, 30] where PM_1_–PM_12_ are 12 PMs for experimental data (exp) and where modeled performance (mod) was based on the parameters (*α*, *β*, *ε*_spr_, *ε*_end_, and *b*) per individual, and $\left( {\sigma _i^{\rm{exp}}} \right)^2$ was the variance in the experimental data of PM_*i*_.

A Monte Carlo-like stochastic search was used for parameter estimation. Our approach ensured that our search explored the parameter space sufficiently, whilst also leading to considerably accurate and reliable parameter values (see Supplementary Methods for more details on parameter generation distributions for the estimation of different model parameters).

## Results

Following participants’ recruitment and random allocation to one of the two experimental conditions (i.e., performance in either isolation or competition; see Supplementary Methods), we verified that the two groups did not differ for key personality traits (i.e., trait anxiety, dominance motivation, competitiveness, self-perceived social rank, and trait physical fatigue), states (i.e., state anxiety and state physical fatigue) or other anthropomorphic characteristics (Supplementary Table 1). The two groups were also equivalent for their levels of physical activity or body mass index, and had similar levels of hand-grip maximal voluntary contraction (MVC) (Supplementary Table 1).

### Glutamate, glutamine, and GABA levels in the nucleus accumbens

We applied ^1^H-MRS to measure metabolite concentrations in the NAc (Fig. 1a; Supplementary Fig. 1). Enhanced spectral resolution, due to performing the neurochemical profiling at 7 T, led to more reliable measurements of J-coupled metabolites, such as Gln, Glu, and GABA, than achievable with ^1^H MRS at lower magnetic fields. Our approach reliably distinguished the methylene groups of Glu and Gln at 2.34 and 2.44 ppm (Fig. 1a). The concentration of metabolites of interest (Glu, Gln, and GABA) and their ratios (Gln/Glu and GABA/Gln) are reported in Fig. 1b and did not differ a priori between experimental groups (Supplementary Table 2). As expected, we found that total accumbal Glu and Gln—and consequently Gln and the Gln/Glu ratio—were highly correlated (Supplementary Table 3). Moderate correlations between Glu and Gln, on the one hand, and GABA were also observed (Supplementary Table 3).

### Competitive context and incentive size enhance task performance

Following ^1^H-MRS acquisition, participants were given the opportunity to work toward different monetary reward levels by squeezing a handgrip at 50% of their MVC (Fig. 1c, d). Given that the neurochemical data from ^1^H-MRS is not time resolved (i.e., a baseline or resting state measurement is taken just before task performance), effort requirements (i.e., handgrip force) were maintained constant across different incentives. This allows assessing performance depending on incentive level without the confounding of divergent effort exertion across participants that is inherent to effort-based decision-making paradigms [1]. The ability to maintain responses over time is a fundamental feature of motivational processes [22], while fatigue from effort exertion tends to impair endurance performance. Accordingly, our task is quite demanding, involving 80 trials distributed across 4 sessions of 20 trials each, enabling to address performance dynamics with time. Moreover, given the importance of breaks for effortful performance [37] we introduced a 3-min break between sessions 2 and 3 to assess individual’s capacity to recover from rest. Finally, we compared performance under competition (*n* = 12) versus self-motivated performance (*n* = 15). For participants in the former group, the monetary gain depended on whether participants’ performance was better or worse than that of a “given” competitor.

Our results indicated that performance was influenced by both social context (*F*_(1, 75)_ = 13.91; *p* = 0.0004) and incentive size (*F*_(2, 75)_ = 12.23; *p* < 0.0001). Thus, competitive context enhanced success rate, and so did incentive size (Fig. 1e; for full data by session and incentive, see Supplementary Fig. 2). There was no interaction between social context and session number (*p* = 0.827; Fig. 1f).

### NAc glutamine relates to better performance and reduced effort perception

**Fig. 2:**
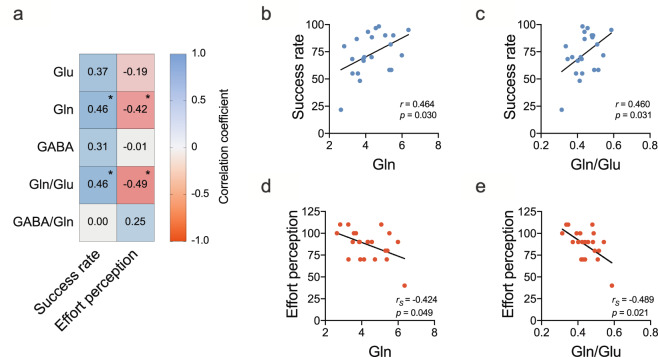
Bivariate correlations between Glu, Gln, GABA, Gln/Glu, and GABA/Gln and total success rate plotted for both groups (isolation and competition) combined. **a** Correlation matrix of Glu, Gln, GABA, Gln/Glu, GABA/Gln with performance and effort perception. **b** NAc glutamine plotted against performance. **c** NAc Gln/Glu plotted against performance. **d** NAc glutamine plotted against effort perception. **e** NAc Gln/Glu plotted against effort perception. Glu glutamate, Gln glutamine, GABA gamma-aminobutyric acid, NAc nucleus accumbens. Success rate was quantified in percentage of correct trials. Effort perception was defined as the level of subjects’ perceived MVC threshold necessary for successful trial completion as described in “Methods”. *p < 0.05. All correlation coefficients are Pearson’s, unless indicated otherwise (Spearman’s; S). *n* = 22.

Before dissecting general performance into its components with computational modeling, we carried out a first set of correlational analyses on all participants (Fig. 2a). We found positive correlations between Gln and Gln/Glu, respectively, and success rate (Fig. 2b, c), while negative correlations between Gln and Gln/Glu, respectively, and effort perception (Fig. 2d, e).

### Computational modeling

We applied computational modeling (see Supplementary Methods for more information) to reveal more intricate components of task performance and, subsequently, to study their relation to the measured NAc metabolite (i.e., Glu, Gln, and GABA) concentrations and their ratios, as well as performance within experimental groups.

### Parameter estimation and model comparison

Briefly, our model consisted of parameters related to utility curvature (*α*, controlling subjective perception of incentive sizes), effort cost (baseline *b* and stamina parameters indicating increased effort cost due to fatigue) and performance function (with steepness *β* of the sigmoidal relationship between outcome utility and success rate). Effort cost baseline b was indicative of overall initial performance, with lower values denoting better performance. Stamina function controlled the loss of energy due to fatigue: sprint stamina (*ε*_spr_) over adjacent sessions (i.e., 2 versus 1 and 4 versus 3), and endurance stamina (*ε*_end_) over the two blocks of the experiment, that were separated by a 3 min break. The stamina functions in our model is a variation from a generic fatiguability function used in other models (e.g., [36]; also see Methods), using two parameters to approximate its rate and recovery during the break. We compared it to a more standard single parameter-based formalization, which performed worse. First, we addressed the contribution of each parameter to performance success throughout the four sessions in the task and for the three different incentives (CHF 0.2, 0.5, and 1). Model simulation results where each parameter is varied separately (Supplementary Fig. 3) show a variety of nonlinear effects that arise due to a sigmoidal relationship between utility and success rate: differences in effort cost baseline (*b*) lead to the largest variability in performance, whereas other parameters have more nuanced effects, limited to certain incentives or experimental sessions.

First, we verified that five parameters were an appropriate model size, capturing the dynamics in individuals’ task performance. For this, we characterized participants’ behavior in the incentive delay task by twelve PMs, relating to the success rates in the different sessions (1–4) and for the different incentive sizes (CHF 0.2, 0.5, and 1). Because these success rates were correlated among each other, we performed a principal component analysis (PCA) to determine the effective dimensionality for the twelve success rate performance measures. The PCA revealed components with gradually decreasing eigenvalues, where the first 5 components explained 85% of the variance (Supplementary Table 4). Although 15% of the variance remained unexplained, it suggested that a model with 5 parameters would be sufficiently flexible to fit performance success adequately. A *χ*^2^ test with *v* = 12 − 5 = 7 degrees of freedom evaluated the goodness of the parameter estimation. Goodness-of-fit error was lower than *χ*^2^_7, 0.05_ = 14.07 for every participant, suggesting that the difference between model fit and experimental data was not significant (*p* > 0.05), hence the model was able to approximate individual performance with a best fitting set of parameters for each participant. Mean goodness of fit error was *χ*^2^ = 3.55 with *p* = 0.83, indicating an excellent fit. Spearman correlation coefficients between the top 4000 estimated parameter sets for each parameter ranged between 0.97 and 0.98, which indicated that our stochastic parameter estimation procedure was stable and reliable. For this reason, applying the best fitting parameter set in statistical analyses seemed appropriate. We also performed parameter recovery analysis by generating performance measures based on model with known parameters and estimating them using the same procedure. Newly estimated and original parameters were highly correlated with each other: Spearman *ρ*(*α*) = 0.963, *ρ*(*β*) = 0.956, *ρ*(*b*) = 0.979, *ρ*(*ε*_spr_) = 0.981, *ρ*(*ε*_end_) = 0.995, indicating high reliability of parameter recovery.

We then compared our model with a simpler formalization of fatiguability, that has a single rate for energy loss (e.g., *ε*_spr_). This corresponds to *ε*_end_ = *ε*_spr_^2^ in our model, in which case energy levels in 4 sessions are proportional to 1, *ε*_spr_, *ε*_spr_^2^, and *ε*_spr_^3^. We performed the identical parameter estimation procedure for this model as for the original model and found that its mean goodness-of-fit *χ*^2^ = 4.343 was significantly worse than of the initial model (paired *t* test *p* = 0.048), which remained true even if smaller number of parameters was considered—4.343 corresponded to *p* = 0.82 of *χ*^2^ distribution with *v* = 12 − 4 = 8 degrees of freedom. Hence, we decided to keep the more flexible model.

### Performance parameters in the isolation and competition groups

We then compared performance parameters between the experimental groups. Participants’ utility curvature (*α*), sprint stamina (*ε*_spr_), and endurance stamina (*ε*_end_) were not normally distributed, whereas those of sigmoidal steepness (*β*) and effort cost baseline (*b*) were (Table 1).

**Table 1:** Comparison of performance parameters between the isolation and competition groups.

Parameter	Normality based on total sample	Group	M/median; SD/IQR	Test statistic and *p* value
*α* (utility curvature)	*p* < 0.0001	Isolation	Median = 0.288 IQR = 1.55	Ranksum = 231 *p* = 0.317
		Competition	Median = 0.102 IQR = 0.688	
*β* (sigmoidal steepness)	*p* = 0.35	Isolation	M = 16.1 SD = 13.9	*t* = 1.89 *p* = 0.0697
		Competition	M = 7.5 SD = 8.0	
*b* (effort cost baseline)	*p* = 0.53	Isolation	M = 0.356 SD = 0.535	*t* = 4.00 *p* = 0.00049
		Competition	M = −0.813 SD = 0.962	
*ε*_spr_ (sprint stamina)	*p* = 0.0032	Isolation	Median = 0.969 IQR = 0.061	Ranksum = 255 *p* = 0.0284
		Competition	Median = 0.621 IQR = 0.87	
*ε*_end_ (endurance stamina)	*p* = 0.013	Isolation	Median = 0.998 IQR = 0.055	Ranksum = 231.5 *p* = 0.284
		Competition	Median = 0.941 IQR = 0.408	

Normality was computed with the Kolmogorov–Smirnov test. M mean, SD standard deviation, IQR interquartile range. Comparisons were computed with Student’s *t* test for normally distributed parameters and with the Mann–Whitney *U* test for not normally distributed parameters.

Participants in the competition group had a substantially lower effort cost baseline (*b*) than in the isolation group (Table 1, two sample *t* test, *t* = 4.00, *p* = 0.00049), indicating higher initial performance success in the competition context. Furthermore, participants in the isolation group exhibited higher sprint stamina than in the competition group (Table 1, Mann–Whitney *U* test, ranksum = 255, *p* = 0.0284), suggesting that the competition context resulted in fatigue quicker than the isolation context.

Correlations between parameters (Supplementary Table 5) were low to moderate, with only sprint and endurance staminas (*ε*_spr_ and *ε*_end_) showing high correlation, indicating partially overlapping neurobehavioral mechanisms. We also tried to fix values of one of these parameters to their average population value and estimated the remaining parameters to see if model fit could be improved (considering lower model complexity). However, with one parameter fixed these models performed considerably worse than the initial model, mean *χ*^2^ (with *ε*_end_ fixed) = 4.82 = *χ*^2^_8, 0.78_ (paired *t* test *P* = 0.020) and *χ*^2^ (with *ε*_spr_ fixed) = 4.67 = *χ*^2^_8, 0.79_ (*P* = 0.021), suggesting that despite high correlation between them, keeping both parameters flexible was essential for the best fit.

Given that anxiety can affect competitive confidence and motivated performance [21, 38], we examined whether anxiety trait is related to the model parameters. We computed associations with measures of state and trait anxiety, and found none of them to be significant (Supplementary Table 6).

### Performance parameters and Gln/Glu ratios

As Gln/Glu concentration ratio was the most strongly correlated with both performance and effort perception (Fig. 2), we examined associations between Gln/Glu and estimated model parameters in order to explore neurochemical correlates of specific components underlying motivated performance (note, however, that associations with all metabolites are provided for reference in Supplementary Table 7). We then performed multiple linear regression with the metabolite concentration ratio and experimental group (i.e., isolation versus competition) as the independent variables and model parameter as the dependent variable. For model parameters that were not normally distributed, rank regression was used. These analyses revealed that Gln/Glu predicted participants’ endurance stamina *ε*_end_ (*p* = 0.003, Table 2) and to a lesser extent their sprint stamina *ε*_spr_ (*p* = 0.010, Table 2). No statistically significant associations were found between Gln/Glu in the occipital lobe and any of the model parameters (Supplementary Table 8).

**Table 2:** Multiple linear regression results (p values) with nucleus accumbens Gln/Glu concentration ratios and experimental task framing as independent variables and estimated model parameter as the dependent variable.

Model parameter	Association with Gln/Glu (*p* values)	Association with task framing (*p* values)
*α* (utility curvature)	0.13	(0.046)
*β* (sigmoidal steepness)	0.29	(0.096)
*b* (threshold baseline)	0.26	(0.008)
*ε*_spr_ (sprint stamina)	*0.010*	(0.045)
*ε*_end_ (endurance stamina)	*0.003*	(0.29)

For negative associations *p* values are shown in parentheses.

We then tested whether differences in average task success rate as well as in model parameters associated with the social context (effort cost baseline b and sprint stamina *ε*_spr_) were mediated by Gln/Glu concentration ratios. For this purpose, we divided participants into those having below versus above average Gln/Glu values and performed a two-way ANOVA with interactions (for not normally distributed *ε*_spr_ using ranks) to study how social context (i.e., isolation versus competition) and Gln/Glu concentration ratios interact in influencing task success rate and performance parameters. We also performed multiple linear regression (for not normally distributed *ε*_spr_ using ranks) with the metabolite concentration ratio, social context (i.e., isolation versus competition) and multiplicative interaction between them (with social context coded −1/1) as the independent variables and task success rate, parameters *b* and *ε*_spr_ as the dependent variables (Supplementary Fig. 4).

**Fig. 3:**
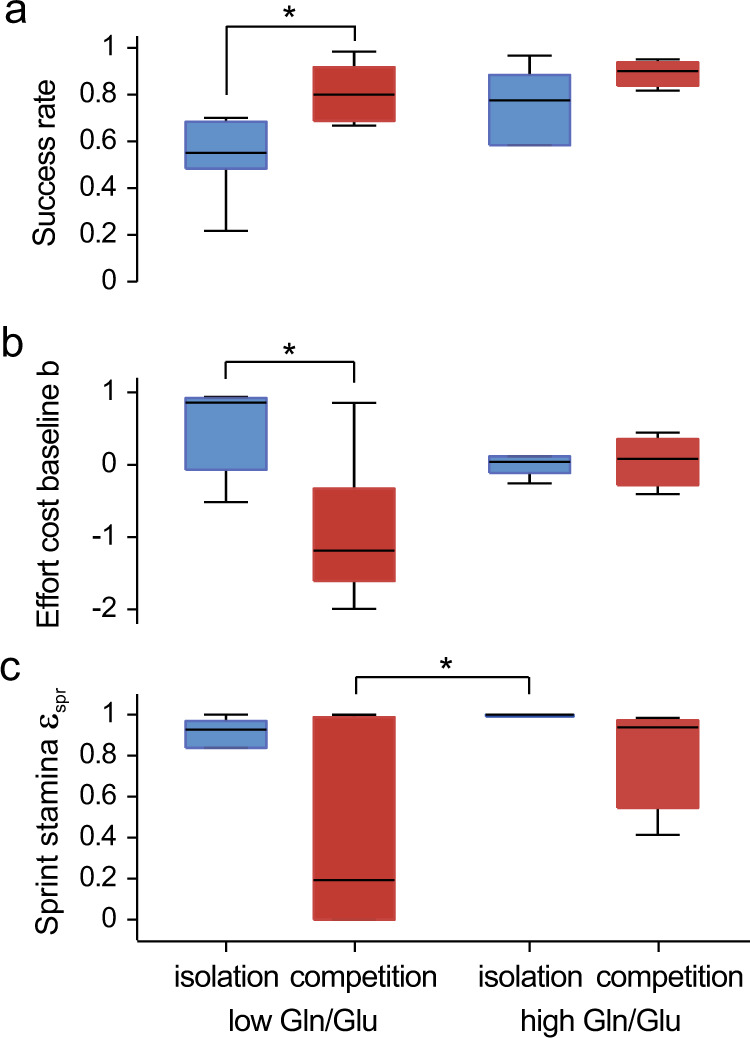
Individual differences in task performance and model parameters as a function of either low or high Gln/Glu ratio in the NAc and social context. Interaction between Gln/Glu ratio and social context (isolation versus competition group) in determining **a** mean overall success rate, **b** effort cost baseline b, which is indicative of initial task performance and **c** sprint stamina ε_spr_, indicative of performance decline over consecutive sessions. Social context only influenced initial performance in participants with low Gln/Glu ratio values, letting them achieve the best performance in the competition condition, despite faster performance decline. Blue bars: isolation; red bars: competition. *p < 0.05.

We found that overall performance (Fig. 3a) was significantly related to both the social context (*F*_1,21_ = 9.3, *p* = 0.007) and Gln/Glu group (*F*_1,21_ = 5.7, *p* = 0.028), with both competition and high Gln/Glu predicting better performance. For effort cost baseline *b* (Fig. 3b), we observed both a significant effect of the social context (*F*_1,21_ = 5.7, *p* = 0.029) and a significant interaction with Gln/Glu group (i.e., high or low) (*F*_1,21_ = 4.5, *p* = 0.048). Further inspection revealed that competition only led to lower *b* values (hence boosted initial performance) in participants with low Gln/Glu values (two sample *t* test *df* = 11, *t* = 3.1, *p* = 0.011), but had no effect on participants with high Gln/Glu ratios (*df* = 7, *t* = 0.28, *p* = 0.79). For sprint stamina *ε*_spr_ (Fig. 3c), there were no significant group effects for the social context (*F*_1,21_ = 3.0, *p* = 0.098) or Gln/Glu group (*F*_1,21_ = 2.8, *p* = 0.11) in ANOVA but a significant positive correlation with Gln/Glu in the regression model (Supplementary Fig. 4). We also found a significant difference between low Gln/Glu group under the competition condition and high Gln/Glu group under the isolation condition (Wilcoxon rank sum test *p* = 0.047, ranksum = 36). This suggests that participants with low Gln/Glu ratio in the competition condition started out the best (low effort cost baseline *b*), but their performance also declined the fastest (low sprint stamina *ε*_spr_).

## Discussion

The nucleus accumbens is part of the brain circuitry that regulates effort-related motivated functions [7, 8, 13]. Here, we investigated whether variation in NAc metabolite levels relate to individual differences in motivated performance. We found that accumbal Gln-to-Glu ratio at resting state predicts the overall average performance in an effort-based motivated task. Importantly, as motivated performance is quite multifaceted, with its different components possibly related to Gln-to-Glu ratio in different ways, we applied computational modeling to deconstruct global performance into specific components. We used a model with its parameters determining curvature of the utility function, sprint and endurance stamina, effort cost baseline and performance randomness, and then estimated the best fitting parameters to each subject’s performance. Subsequently, we inquired which performance components are better predicted by accumbal metabolites. This approach allowed us to relate individual differences in accumbal Gln-to-Glu ratio specifically with variation in endurance stamina (*ε*_end_, the capacity to recover performance following a short rest-break period) and—to a lesser extent—sprint stamina (*ε*_spr_, a measure of fatigability) displayed by participants subsequently, during motivated performance.

The neurochemical profiling in this study benefitted from the ability of increased spectral resolution at 7 T to reliably measure J-coupled metabolites, such as Gln, Glu, and GABA separately. This is an advantage over substantial prior work performed at ≤3 T that due to limitations to separate Glu and Gln, frequently report Glx as the compound measurement of these two metabolites. Glutamine is synthesized from Glu in astrocytes in a reaction catalyzed by Gln synthetase [39]. Once astrocytes release Gln, it can be taken up by neurons where it is readily converted to Glu [39, 40] (in GABAergic neurons, Glu is further converted into GABA [41]). Therefore, obtaining the distinct measurement of Gln is very important as it allows estimating the ratios between Gln and, respectively, Glu and GABA, thus providing indices of neurotransmitter potential or “reservoir”.

Our computational model-based analysis approach, which has been successful in elucidating internal behavioral variables of reward-based learning (such as learning rates [42], exploration–explotation balance [30], discounting [43], episodic memory [29], effort [14], mood [44], social [45], and economic preferences [46, 47]), allowed us here to provide key insights into the specific components of motivated performance that relate to accumbal metabolites. We found that the Gln-to-Glu ratio is positively linked to stamina necessary to maintain performance over longer periods, whereas the competition context leads to substantially improved initial performance accompanied by a faster loss in stamina, and which was particularly pronounced in individuals with low resting Gln-to-Glu levels. Importantly, our data suggests that Gln rather than Glu or GABA is the main contributor to the reported association between accumbal Gln-to-Glu ratio and endurance stamina (*ε*_end_).

Medium spiny neurons, the main neuronal type in the nucleus accumbens comprising about 95% of accumbal cells, range from GABAergic to mixed GABAergic and glutamatergic [48]. In addition, the nucleus accumbens receives GABAergic and glutamatergic projections from multiple brain regions [48, 49]. Therefore, Glu and GABA concentrations measured in the NAc through ^1^H-MRS represent pools of both accumbal and afferent neurons. Due to the more local nature of glial cells, Gln levels in our data primarily represent production by accumbal glial cells (including astrocytes and oligodendrocytes) [50, 51]. Therefore, our findings point towards a key contribution of accumbal glial-derived Gln, and particularly the Gln-to-Glu ratio, on effortful endurance in motivated behavior.

It is important to note that, in addition to its role in neurotransmitter production, Gln is also involved in mitochondrial oxidative phosphorylation and, consequently, cellular energy production [20, 52]. Therefore, high accumbal Gln levels (particularly in situations of increased Gln-to-Glu ratio) can be regarded as a multifunctional metabolic reservoir that, depending on cellular requirements, can be readily utilized for neurotransmitter production and/or fueling mitochondrial function [53, 54]. Accordingly, high Gln concentrations might allow for enhanced GABA and Glu concentrations to be achieved (via just-in-time synthesis) during task performance [55], accommodating the behavioral requirements to succeed. Indeed, our results suggest that performance is not maintained via already present GABA or Glu pools, because neither resting-state GABA nor Glu predicted endurance performance.

In addition, high Gln concentrations may contribute to fuel mitochondrial function under enhanced NAc engagement during motivated and effortful behaviors, which are recognized accumbal putative actions [10, 13, 56, 57]. In support of this hypothesis, work in rodents has shown that the capacity to remain on task for longer and to overcome greater effort costs is related to the magnitude of NAc oxygen responses to delivered rewards [58]. Oxygen consumption reflects mitochondrial respiration, a critical mitochondrial function and partial proxy for energy production in the form of ATP. Strikingly, higher mitochondrial oxygen respiratory capacity in the NAc in male rodents is positively related to the capacity to win a social competition [59, 60]. Therefore, our results relating accumbal Gln-to-Glu ratio to the capacity to exert motivated effort provide novel metabolic insights to this body of data.

One of the mechanisms whereby Gln-to-Glu ratio may contribute to differences in endurance and sprint stamina is the capacity of individuals with a high Gln-to-Glu ratio to overcome fatigue. Fatigue induced by prolonged active task engagement can have a negative impact on endurance performance. Performance decrements induced by fatigue have been proposed to be due to perception of effort and can lead to task disengagement or “giving up” [61]. In sports, for example, the psychobiological model of endurance performance states that effort perception plays a crucial role in explaining how fatigue reduces the willingness to perform [62] and negatively affects performance [61]. Recent accounts of human motivated performance emphasize the perception of effort as a crucial factor underlying motor endurance performance [63]. The inverse relationship that we found between Gln-to-Glu levels and effort perception may provide support for this account, especially in light of our decomposition of motivated performance into an early component that is boosted in low Gln-to-Glu individuals under competition (as a sign of excessive effort) and the stamina component, which is, subsequently, reduced in the same individuals. Although, to the best of our knowledge, no previous study assessed NAc metabolism in the context of fatigue, substantial influence relates fatigue with reduced Gln in blood [64] and potentially also with alterations in Gln metabolism in the brain [65]. Oral glutamine supplementation has been reported to reduce subjective fatigue and ratings of perceived exertion during demanding tasks [66, 67] and to increase striatal Gln levels [68].

At the neurobiological level, several mechanisms may account for the observed differences in Gln and Gln-to-Glu ratio, including differences in Gln production, metabolite catabolism and the availability of cellular transporters for Gln and Glu. The nucleus accumbens has been implicated in anhedonia, reduced motivation, and decreased energy typically found in individuals with depression [69] and substantial data indicate alterations in these metabolic pathways and depression. For example, reduced cortical density of glutamine synthetase-expressing astrocytes has been found in post mortem brains of major depression patients [50] and genetic variation in the gene coding for this enzyme associated with depression [70]. In addition, reduced levels in NAc Gln were reported in a rat model of stress-induced depression [44].

We also found that performance in our task was enhanced by social competition, in alignment with a reported facilitative role of competition in task performance [25–28]. Our computational model revealed that the facilitating effect of competition on the overall performance was mostly due to improvement in initial performance (expressed through lower values of effort cost baseline *b*), which persisted despite lower values of sprint stamina (*ε*_spr_, which manifested itself in poorer performance in later task sessions). Furthermore, we found a significant interaction between competition context and Gln-to-Glu ratios in determining initial performance: the initial performance boost triggered by social competition was only observed for participants with a low Gln-to-Glu ratio. Participants characterized by a high Gln-to-Glu ratio seemed to have superior task engagement from the onset of the task, which was not significantly boosted by competition. This observation suggests a link between high accumbal Gln-to-Glu and self-motivated performance in effortful incentivized challenges.

Finally, the associations between metabolites and performance were not observed in the occipital lobe, in agreement with reports indicating a lack of correspondence in Gln or Gln-to-Glu levels across different brain regions [71]. However, a note of caution should be added, as due to technical limitations, metabolites in the occipital lobe were acquired on a different day in a subsample of the participants. This implies that the power to detect a significant behavior–metabolite association in the occipital lobe was lower, and it would be important in future studies to verify the specificity of the reported associations for the NAc. In this study, only males were included as our prediction for a link between accumbal metabolism and motivated performance was inspired in background studies involving male rodents [59, 72, 73]. In addition, due to technical challenges mainly associated with scanning at 7 T, our original participant recruitment was reduced to a moderate sample size, which limits the generalization of the obtained results. Therefore, future studies are warranted to test whether our findings are generalized to the general population. Finally, although our approach allowed us to reveal important components of motivated performance, in the future it will be important to disentangle further aspects such as effort-related decision-making and vigor of response.

In conclusion, our results provide the first solid evidence for the role of accumbal metabolites—particularly the Gln-to-Glu ratio—in different components of effortful performance. We envision that this approach and findings can help developing metabolism-targeting strategies to ameliorate deficits in motivated effort engagement.

## Funding and Disclosure

This work was supported by grants from the Swiss National Science Foundation (CR20I3-146431; NCCR Synapsy grant number 51NF40-158776) and intramural funding from the EPFL. The authors declare no competing interests. Open Access funding provided by EPFL Lausanne.

## Supplementary information

Supplementary Information

## Data Availability

The data that support the findings of this study are available from the corresponding authors upon request.

## Code availability

The code used in this study is available from the corresponding authors upon request.

## References

[1] Chong TT-J, Bonnelle V, Husain M (2016). Quantifying motivation with effort-based decision-making paradigms in health and disease. Prog Brain Res.

[2] Duckworth AL, Eichstaedt JC, Ungar LH (2015). The mechanics of human achievement. Soc Personal Psychol Compass.

[3] Kanfer R, Frese M, Johnson RE (2017). Motivation related to work: a century of progress. J Appl Psychol.

[4] Epstein J, Silbersweig D (2015). The neuropsychiatric spectrum of motivational disorders. J Neuropsychiatry Clin Neurosci.

[5] Admon R, Pizzagalli DA (2015). Corticostriatal pathways contribute to the natural time course of positive mood. Nat Commun.

[6] Zald DH, Treadway MT (2017). Reward processing, neuroeconomics, and psychopathology. Annu Rev Clin Psychol.

[7] Pessiglione M, Vinckier F, Bouret S, Daunizeau J, Le Bouc R (2017). Why not try harder? Computational approach to motivation deficits in neuro-psychiatric diseases. Brain.

[8] Salamone JD, Correa M, Farrar A, Mingote SM (2007). Effort-related functions of nucleus accumbens dopamine and associated forebrain circuits. Psychopharmacology.

[9] Floresco SB (2015). The nucleus accumbens: an interface between cognition, emotion, and action. Annu Rev Psychol.

[10] Knutson B, Adams CM, Fong GW, Hommer D (2001). Anticipation of increasing monetary reward selectively recruits nucleus accumbens. J Neurosci.

[11] Pessiglione M, Schmidt L, Draganski B, Kalisch R, Lau H, Dolan RJ (2007). How the brain translates money into force: a neuroimaging study of subliminal motivation. Science.

[12] Croxson PL, Walton ME, O’Reilly JX, Behrens TEJ, Rushworth MFS (2009). Effort-based cost-benefit valuation and the human brain. J Neurosci Off. J Neurosci.

[13] Schmidt L, Lebreton M, Cléry-Melin M-L, Daunizeau J, Pessiglione M (2012). Neural mechanisms underlying motivation of mental versus physical effort. PLoS Biol.

[14] Hauser TU, Eldar E, Dolan RJ (2017). Separate mesocortical and mesolimbic pathways encode effort and reward learning signals. Proc Natl Acad Sci USA.

[15] Jocham G, Hunt LT, Near J, Behrens TEJ (2012). A mechanism for value-guided choice based on the excitation-inhibition balance in prefrontal cortex. Nat Neurosci.

[16] Hu Y, Chen X, Gu H, Yang Y (2013). Resting-state glutamate and GABA concentrations predict task-induced deactivation in the default mode network. J Neurosci Off. J Soc Neurosci.

[17] Yoon JH, Grandelis A, Maddock RJ (2016). Dorsolateral prefrontal cortex GABA concentration in humans predicts working memory load processing capacity. J Neurosci.

[18] Scholl J, Kolling N, Nelissen N, Stagg A, Harmer CJ, Rushworth MF (2017). Excitation and inhibition in anterior cingulate predict use of past experiences. ELife.

[19] Walls AB, Waagepetersen HS, Bak LK, Schousboe A, Sonnewald U (2015). The glutamine-glutamate/GABA cycle: function, regional differences in glutamate and GABA production and effects of interference with GABA metabolism. Neurochem Res.

[20] Albrecht J, Sidoryk-Węgrzynowicz M, Zielińska M, Aschner M (2010). Roles of glutamine in neurotransmission. Neuron Glia Biol.

[21] Berchio C, Rodrigues J, Strasser A, Michel CM, Sandi C (2019). Trait anxiety on effort allocation to monetary incentives: a behavioral and high-density EEG study. Transl Psychiatry.

[22] Salamone JD, Yohn SE, López-Cruz L, San Miguel N, Correa M (2016). Activational and effort-related aspects of motivation: neural mechanisms and implications for psychopathology. Brain. J Neurol.

[23] Covington MV (2000). Goal theory, motivation, and school achievement: an integrative review. Annu Rev Psychol.

[24] Gilman JM, Treadway MT, Curran MT, Calderon V, Evins AE (2015). Effect of social influence on effort-allocation for monetary rewards. PLoS ONE.

[25] Stanne MB, Johnson DW, Johnson RT (1999). Does competition enhance or inhibit motor performance: a meta-analysis. Psychol Bull..

[26] Cooke A, Kavussanu M, McIntyre D, Ring C (2011). Effects of competition on endurance performance and the underlying psychological and physiological mechanisms. Biol Psychol.

[27] Le Bouc R, Pessiglione M (2013). Imaging social motivation: distinct brain mechanisms drive effort production during collaboration versus competition. J Neurosci.

[28] Kilduff GJ (2014). Driven to win: rivalry, motivation, and performance. Soc Psychol Personal Sci.

[29] Luksys G, Fastenrath M, Coynel D, Freytag V, Gschwind L, Heck A (2015). Computational dissection of human episodic memory reveals mental process-specific genetic profiles. Proc Natl Acad Sci USA.

[30] Luksys G, Gerstner W, Sandi C (2009). Stress, genotype and norepinephrine in the prediction of mouse behavior using reinforcement learning. Nat Neurosci.

[31] Nassar MR, Frank MJ (2016). Taming the beast: extracting generalizable knowledge from computational models of cognition. Curr Opin Behav Sci.

[32] Corrado G, Doya K (2007). Understanding neural coding through the model-based analysis of decision making. J Neurosci.

[33] Strasser A, Xin L, Gruetter R, Sandi C (2019). Nucleus accumbens neurochemistry in human anxiety: a 7 T 1H-MRS study. Eur Neuropsychopharmacol.

[34] Neto LL, Oliveira E, Correia F, Ferreira AG (2008). The human nucleus accumbens: where is it? A stereotactic, anatomical and magnetic resonance imaging study. Neuromodul Technol Neural Interface.

[35] Baujard A. Welfare Economics. Rochester. NY: Social Science Research Network; 2013.

[36] Le Bouc R, Rigoux L, Schmidt L, Degos B, Welter M-L, Vidailhet M (2016). Computational dissection of dopamine motor and motivational functions in humans. J Neurosci.

[37] Blasche G, Szabo B, Wagner‐Menghin M, Ekmekcioglu C, Gollner E (2018). Comparison of rest-break interventions during a mentally demanding task. Stress Health.

[38] Goette L, Bendahan S, Thoresen J, Hollis F, Sandi C (2015). Stress pulls us apart: anxiety leads to differences in competitive confidence under stress. Psychoneuroendocrinology.

[39] Schousboe A (2019). Metabolic signaling in the brain and the role of astrocytes in control of glutamate and GABA neurotransmission. Neurosci Lett.

[40] Perea G, Navarrete M, Araque A (2009). Tripartite synapses: astrocytes process and control synaptic information. Trends Neurosci.

[41] Rothman DL, Hyder F, Sibson N, Behar KL, Mason GF, Petroff OA (1999). In vivo magnetic resonance spectroscopy studies of the glutamate and GABA neurotransmitter cycles and functional neuroenergetics. Philos Trans R Soc Lond B Biol Sci.

[42] Frank MJ, Moustafa AA, Haughey HM, Curran T, Hutchison KE (2007). Genetic triple dissociation reveals multiple roles for dopamine in reinforcement learning. Proc Natl Acad Sci USA.

[43] Schweighofer N, Bertin M, Shishida K, Okamoto Y, Tanaka SC, Yamawaki S (2008). Low-serotonin levels increase delayed reward discounting in humans. J Neurosci.

[44] Rappeneau V, Blaker A, Petro JR, Yamamoto BK, Shimamoto A (2016). Disruption of the glutamate-glutamine cycle involving astrocytes in an animal model of depression for males and females. Front Behav Neurosci.

[45] Set E, Saez I, Zhu L, Houser DE, Myung N, Zhong S (2014). Dissociable contribution of prefrontal and striatal dopaminergic genes to learning in economic games. Proc Natl Acad Sci USA.

[46] Hsu M, Bhatt M, Adolphs R, Tranel D, Camerer CF (2005). Neural systems responding to degrees of uncertainty in human decision-making. Science.

[47] Blain B, Schmit C, Aubry A, Hausswirth C, Le Meur Y, Pessiglione M (2019). Neuro-computational impact of physical training overload on economic decision-making. Curr Biol.

[48] Kardos J, Dobolyi Á, Szabó Z, Simon Á, Lourmet G, Palkovits M, et al. Molecular plasticity of the nucleus accumbens revisited—astrocytic waves shall rise. Mol Neurobiol. 2019. 10.1007/s12035-019-1641-z. 10.1007/s12035-019-1641-z PMC6834761 31134458

[49] Britt JP, Benaliouad F, McDevitt RA, Stuber GD, Wise RA, Bonci A (2012). Synaptic and behavioral profile of multiple glutamatergic inputs to the nucleus accumbens. Neuron.

[50] Bernstein H-G, Meyer-Lotz G, Dobrowolny H, Bannier J, Steiner J, Walter M (2015). Reduced density of glutamine synthetase immunoreactive astrocytes in different cortical areas in major depression but not in bipolar I disorder. Front Cell Neurosci.

[51] Xin W, Mironova YA, Shen H, Marino RAM, Waisman A, Lamers WH (2019). Oligodendrocytes support neuronal glutamatergic transmission via expression of glutamine synthetase. Cell Rep..

[52] Mazat J-P, Ransac S (2019). The fate of glutamine in human metabolism. the interplay with glucose in proliferating cells. Metabolites.

[53] McKenna MC (2007). The glutamate-glutamine cycle is not stoichiometric: fates of glutamate in brain. J Neurosci Res.

[54] Rangaraju V, Calloway N, Ryan TA (2014). Activity-driven local ATP synthesis is required for synaptic function. Cell.

[55] Tani H, Dulla CG, Farzampour Z, Taylor-Weiner A, Huguenard JR, Reimer RJ (2014). A local glutamate-glutamine cycle sustains synaptic excitatory transmitter release. Neuron.

[56] Haber SN, Behrens TE (2014). The neural network underlying incentive-based learning: implications for interpreting circuit disruptions in psychiatric disorders. Neuron.

[57] Otis JM, Namboodiri VM, Matan AM, Voets ES, Mohorn EP, Kosyk O (2017). Prefrontal cortex output circuits guide reward seeking through divergent cue encoding. Nature.

[58] Hailwood JM, Gilmour G, Robbins TW, Saksida LM, Bussey TJ, Marston HM (2018). Oxygen responses within the nucleus accumbens are associated with individual differences in effort exertion in rats. Eur J Neurosci.

[59] Hollis F, Kooij MA, van der, Zanoletti O, Lozano L, Cantó C, Sandi C (2015). Mitochondrial function in the brain links anxiety with social subordination. Proc Natl Acad Sci.

[60] van der Kooij MA, Zalachoras I, Sandi C (2018). GABAA receptors in the ventral tegmental area control the outcome of a social competition in rats. Neuropharmacology.

[61] Marcora SM, Staiano W (2010). The limit to exercise tolerance in humans: mind over muscle?. Eur J Appl Physiol.

[62] Massar SAA, Csathó Á, Van der Linden D (2018). Quantifying the motivational effects of cognitive fatigue through effort-based decision making. Front Psychol..

[63] Inzlicht M, Marcora SM (2016). The central governor model of exercise regulation teaches us precious little about the nature of mental fatigue and self-control failure. Front Psychol.

[64] Armstrong CW, McGregor NR, Sheedy JR, Buttfield I, Butt HL, Gooley PR (2012). NMR metabolic profiling of serum identifies amino acid disturbances in chronic fatigue syndrome. Clin Chim Acta.

[65] Wilkinson DJ, Smeeton NJ, Watt PW (2010). Ammonia metabolism, the brain and fatigue; revisiting the link. Prog Neurobiol.

[66] Coqueiro AY, Raizel R, Bonvini A, Hypólito T, Godois A da M, Pereira JRR (2018). Effects of glutamine and alanine supplementation on central fatigue markers in rats submitted to resistance training. Nutrients.

[67] Nava RC, Zuhl MN, Moriarty TA, Amorim FT, Bourbeau KC, Welch AM (2019). The effect of acute glutamine supplementation on markers of inflammation and fatigue during consecutive days of simulated wildland firefighting. J Occup Environ Med.

[68] Wang L, Maher TJ, Wurtman RJ (2007). Oral L-glutamine increases GABA levels in striatal tissue and extracellular fluid. FASEB J.

[69] Nestler EJ, Carlezon WA (2006). The mesolimbic dopamine reward circuit in depression. Biol Psychiatry.

[70] Sequeira A, Mamdani F, Ernst C, Vawter MP, Bunney WE, Lebel V (2009). Global brain gene expression analysis links glutamatergic and GABAergic alterations to suicide and major depression. PloS ONE.

[71] Öngür D, Haddad S, Prescot AP, Jensen JE, Siburian R, Cohen BM (2011). Relationship between genetic variation in the glutaminase gene GLS1 and brain glutamine/glutamate ratio measured in vivo. Biol Psychiatry.

[72] Larrieu T, Cherix A, Duque A, Rodrigues J, Lei H, Gruetter R (2017). Hierarchical status predicts behavioral vulnerability and nucleus accumbens metabolic profile following chronic social defeat stress. Curr Biol.

[73] Cherix A, Larrieu T, Grosse J, Rodrigues J, McEwen B, Nasca C (2020). Metabolic signature in nucleus accumbens for anti-depressant-like effects of acetyl-L-carnitine. Elife.

