# Supplementary material for: Glutamine-to-glutamate ratio in the nucleus accumbens predicts effort-based motivated performance in humans
Source: Neuropsychopharmacology. 2020 Jul 20;45(12):2048–57. doi: 10.1038/s41386-020-0760-6 (PMC7547698; doi:10.1038/s41386-020-0760-6)
Supplement: Supplementary file 1 — Supplementary Information [file 41386_2020_760_MOESM1_ESM.docx]

**Supplementary Material**

**Glutamine-to-glutamate ratio in the nucleus accumbens predicts effort-based motivated performance in humans**

Alina Strasser, Gediminas Luksys, Lijing Xin, Mathias Pessiglione, Rolf Gruetter, Carmen Sandi

**Supplementary Methods**

## Participants

We obtained approval for this study from the Cantonal Ethics Committee of Vaud, Switzerland. Forty-three healthy male individuals from the Ecole Polytechnique Fédérale de Lausanne (EPFL) and the University of Lausanne were recruited for the study. Informed written consent was obtained from all participants before participating and participants were debriefed after the experiment. All experiments were performed in accordance with the Declaration of Helsinki. Phenotypic and ^1^H-MRS data was collected on two different days for the two brain regions. First, we collected NAc ^1^H-MRS data (n = 27), and then on a separate day (1-4 weeks after the NAc measurements) we collected the data from the occipital lobe (OL; n = 17). Obtaining reliable ^1^H-MRS data from the NAc is highly demanding, and requires around 90 min of scan time. Further, out of the initially recruited 43 participants, data from 16 participants could not be fully collected and included in the analysis due to the following reasons: 4 participants dropped out voluntarily on the scanning day, 4 participants reported metal implants only on the scanning day, 2 participants were not suited for the scanner environment due to their anthropometrics, 4 participants moved during neuroimaging and for 2 participants hardware failed during acquisition. Whilst metabolite data was available for 27 individuals, we excluded behavioral data for 5 out of those (2 due to hardware failure and 3 due to task non-compliance). Thus, our brain-behavior analyses are based on 22 subjects. Out of these, 17 participants were successfully recruited for the second OL scan, and these 17 serve as the experimental control group for the neurochemical assessment.

Inclusion criteria for the study were being male, 20-30 years old, right-handed, no regular drug or medication intake, non-smoker, no history of psychiatric or neurological illness, and no metallic implants.

All experiments were performed between noon and 6 pm. Participants were instructed not to eat or drink any caffeinated drinks one hour before the experiment, and not to be hungry or thirsty when arriving in the laboratory. They were also instructed not to take any medication and to avoid physical effort within 24 hours before the experiment. These study instructions were verified by the experimenter before each experiment started.

## Personality questionnaires and anthropometric characteristics

We assessed state and trait anxiety with the State-Trait Anxiety Inventory (STAI) [1], social dominance with the Personality Research Form (PRF) dominance scale which measures dominance motivation [2], competitiveness with the revised competitiveness index capturing interpersonal competitiveness in everyday contexts[3], self-perceived social rank with the social comparison scale [4], participants’ physical fatigue (state and trait) and physical activity with the Mental and Physical State Energy and Fatigue Scales (SEF) [5]. In addition to the above scales, we measured age, height, weight, computed the BMI and elicited individuals’ maximal voluntary contraction (MVC), the latter procedure is described in the Methods below. These questionnaire items and anthropometric details were used for optimal group matching between participants' characteristics in the isolation and competition condition. Supplementary Table 1 summarizes these personality and anthropometric characteristics, and shows that the two groups were optimally matched.

## Proton Magnetic Resonance Spectroscopy (^1^H-MRS) acquisition and data processing

The MR measurements were performed on a Magnetom 7T/68-cm head scanner (Siemens, Erlangen, Germany) equipped with a single-channel quadrature transmit and a 32-channel receive coil (Nova Medical Inc., MA, USA). MR images were acquired with a magnetization-prepared rapid gradient-echo (MP2RAGE) sequence for MRS voxel positioning with the following parameters: repetition time (TR) = 5500 ms, echo time (TE) = 1.87 ms, inversion time (TI)_1_ = 750 ms, TI_2_ = 2350 ms, α_1_ = 4°, α_2_ = 5°, 1 × 1 × 1 mm resolution, matrix size = 210 × 210 × 160 [6].

The NAc region of interest voxel (VOI) was defined by the third ventricle medially, the subcallosal area inferiorly, and the body of the caudate nucleus and the putamen laterally and superiorly, in line with definitions of NAc anatomy identifiable on MRIs [7] (Supplementary Fig. 1).

Magnetic field inhomogeneities within the VOI were minimized using 1^st^- and 2^nd^-order shims with the fast, automatic shim technique using echo-planar signal readout for mapping along projections FAST(EST)MAP sequence [8,9].

^1^H-MR spectra were acquired with the semi-adiabatic spin-echo full-intensity acquired localized (semi-adiabatic SPECIAL) sequence [10] in both the NAc (VOI = 14 × 10 × 13 mm^3^, TR/TE = 6500/16 ms, bandwidth = 4000 Hz, vector size = 2048 pts, average of 256) and the OL (VOI = 25 × 20 × 20 mm^3^, TR/TE = 8000/16 ms, bandwidth = 4000 Hz, vector size = 2048 pts, average of 64) including 6 outer volume suppression bands and water suppression using the variable pulse power and optimized relaxation delays (VAPOR) sequence. The unsuppressed water signal was acquired and used as an internal reference for the metabolite quantification and eddy current correction.

Localized single-voxel ^1^H-MR spectra from the left accumbens were acquired in twenty-seven participants. ^1^H-MR spectra from the bilateral occipital lobe were acquired as the experimental control on a different day in the participants that were recruited to return to the laboratory (n = 17). All ^1^H-MR spectra were frequency corrected, summed and then analyzed with LCModel with a basis set including simulated metabolite spectra and an experimentally measured macromolecule baseline [11–13]. MR images were segmented and grey matter (GM), white matter (WM) and cerebrospinal fluid (CSF) percentages inside the MRS voxel were evaluated [14] and used to calculate water concentration assuming water concentrations of 43300 mM in GM, 35880 mM in WM, and 55556 mM in CSF [15]. Metabolite concentrations were then partial-volume corrected for the CSF fraction.

A representative spectrum of the NAc voxel is shown in Figure 1a. We obtained an overall spectral SNR and linewidth of 72 ± 9 and 0.048 ± 0.006 ppm, respectively. Glu, Gln and GABA concentrations in the NAc were quantified with CRLB of 2.36 ± 0.49 %, 4.91 ± 0.75 % and 11.32 ± 2.78 % (Supplementary Table 2). The signal-to-noise ratio (SNR) was calculated using the peak height of *N*-acetyl-aspartate (NAA) at 2.01 ppm divided by the SD of the noise between 9.5 - 10 ppm, where LCModel provided the FWHM (ppm). Metabolite concentrations of Glu, Gln and GABA are reported in μmol/g.

## Effort-related Monetary Incentive Force Task

Our modified MID version [16] relied on exerting force on a hand grip or dynamometer (TSD121B-MRI, Biopac) at a threshold corresponding to 50% of each participant’s maximum voluntary contraction (MVC) and, therefore, was termed monetary incentive force (MIF) Task. We set this individual task threshold before the ^1^H-MRS data acquisition by instructing each participant to exert as much force as possible on the dynamometer (i.e., a hand grip, see Figure 1d) for 1 sec. We repeated this procedure 3 times interspersed by breaks of 3 min each to allow for recovery. The highest out of the 3 values was used to calibrate the threshold for the handgrip force required to succeed in the MIF task. During behavioral assessment, force (in kilogram, kg) was continuously recorded in Acknowledge 4.3 (BIOPAC Systems, United States). Visual signal inspection confirmed the absence of artefacts.

For the behavioral assessment, participants were comfortably seated in front of a computer screen at 90 cm distance and were instructed to keep the same right upper limb position (i.e., upper arm and forearm at 90° angle and hand extended) whenever using the dynamometer. The dynamometer signal, linearly proportional to the exerted force, served as a real-time input to the PC that delivered the MIF task (E-Prime software). The task comprised a total of 80 trials that were run in 2 blocks with a 3 min break between the 2 blocks. Each block contained 2 sessions and each session had twenty trials: 5 incentivized trials of each of the different incentives (0.2, 0.5, 1) presented in a random order, and 5 non-incentivized rest trials occurring after every 3 incentivized trials. To earn the displayed incentives, the participant’s 50% MVC threshold had to be reached within 2 sec and the force at or above the threshold maintained for another 3 sec. Performance was guided by visual cues on the screen (Fig. 2c, d) that were adapting to participants' performance in real-time.

During the task, trials started with a fixation cross (varying between 1-4 sec), followed by an anticipatory signal (3 sec) indicating the trial’s incentive. To earn the monetary incentives, participants were instructed to exert force on the dynamometer. The beginning of the force exertion period was signaled by the appearance of a red circle around the fixation cross. If the established threshold (i.e., 50% of the participant’s MVC force) was reached within 2 sec, the red circle was replaced by a green circle. The green circle also indicated that participants had to maintain the contraction force level above the threshold for 3 more seconds. If participants did not reach the threshold in the initial 2 sec or if the force level fell below the maintenance threshold during the 3 sec maintenance period, the trial was failed, and visualized by a red cross occurring on the screen. If the force was maintained for the required 3 sec, a green tick indicated successful task performance during a single trial (see Fig. 1c and d for task details). Total trial duration was fixed to 10 sec. Before entering the scanner environment, participants performed 1 pre-training session of 20 trials (as described above) during which no incentives could be earned.

To investigate the influence of competition on performance, the experiment was run under two experimental conditions, an isolation and a competition condition, both requiring the same individually calibrated 50% of MVC threshold and most experimental conditions being equal. In both groups, participants were informed that one out of the four sessions would be randomly drawn at the end of the experiment, and that they would be paid according to their performance in that particular session (i.e., they would be paid the coins that were earned in this session). Furthermore, they were told that one additional session would be drawn randomly and they would also be paid according to their performance in this session under the following condition: (1) For participants performing in the isolation context: if a coin that will be tossed at the end of the experiment will land on heads or (2) For participants performing in the competition context: if they performed overall more successful trials than another participant of the experiment (that had already performed the task) and to which they had been paired (having similar age and physical strength as the participant). The payment for each participant was computed accordingly at the end of each experimental session and each participant was immediately paid.

Therefore, in both experimental contexts (i.e. isolation and competition), half of the expected monetary outcome was dependent on either a coin toss (participants performing in ‘isolation’) or another participant’s performance (participants performing in ‘competition’). Thus, whilst the objective probability to obtain half of the expected monetary outcome was equivalent in both contexts, the subjective probability was performance-dependent in the competition context.

Success was computed in % of successful trials out of total trials, and for each of the four sessions (i.e., Success_Total_, Success_Session 1_, Success_Session 2_, Success_Session 3_, Success_Session 4_) and for each of the three incentives (i.e., CHF 0.2, 0.5 and 1).

*Effort perception*

We measured effort perception for each participant after the MIF task. For this purpose, at the end of the experiment, each participant was asked to estimate the threshold at which their force was successful to activate the green circle during the experiment, on a scale from 10% to 120% of their MVC, with steps of 10% (note that the actual threshold was set at 50%). This single measurement after the MIF task was the basis for our association analyses between effort perception and NAc metabolites.

**Estimation and evaluation of best-fitting model parameters**

Twelve performance measures (PMs) were chosen: success rates in each of the 4 sessions and for each of the 3 incentives. To evaluate how well the model fits participants’ performance, the following goodness of fit function was used, with

$\chi^{2}=\sum_{i=1}^{12} \frac{{{({PM}_{i}^{exp}- PM}_{i}^{mod})}^{2}}{\left( \sigma_{i}^{exp} \right)^{2}}$ (1.4)

where PM_1_ to PM_12_ are 12 PMs for experimental data (exp) and where modeled performance (mod) was based on the parameters (α, β, ε_spr_, ε_end_, and *b*) per individual, and $\left( \sigma_{i}^{exp} \right)^{2}$ was the variance in the experimental data of PM_i_.

A Monte Carlo-like stochastic search was used for parameter estimation. Per individual, 16000000 random parameter sets were generated based on the distributions described below. 4000 parameter sets were then selected based on the smallest $\chi^{2}$. Then, for each of the 4000 selected sets we generated 4000 new sets by adding the parameters of the selected sets with random parameters based on the initial distributions but with 1/5 of their range (e.g., if the initial distribution was a normal distribution with mean 1.5 and a SD of 1, the added steps had then a mean of 0 and SD of 0.2). This enhanced the local search around the best fitting parameters identified initially. Finally, 4000 parameter sets containing the smallest $\chi^{2}$ (which decreased compared to the first iteration) were selected and for each of them 4000 new sets were generated by adding the parameters of the selected sets with random parameters based on the initial distributions but with 1/25 of their range, which refined the search further. This approach ensured that our search explored the parameter space sufficiently, whilst also leading to considerably accurate and reliable parameter values (see Supplementary Methods for more details on parameter generation).

The initial distributions for the estimation of different model parameters were the following: α – exp of normal distribution with mean -1.5 and SD 2; β – exp of normal distribution with mean 1.5 and SD 1; ε_spr_ and ε_end_ – square root of uniform distribution between 0 and 1; *b* – normal distribution with mean 0 and SD 1. These distributions were chosen with constraints of the model in mind (i.e., that α and β were both positive and α values were to fall below 1 to reflect concave utility) as well as after initial experimentation to ensure efficiency of parameter estimation and that few estimated values fall at the ends of the search ranges. However, they did not critically affect parameter estimation results. Even with different distributions our estimation produced similar, but noisier and sometimes less consistent, parameter values.

## Statistical analyses

Data was processed and analyzed in IBM SPSS Statistics 20, MATLAB R2017a, and GraphPad Prism 8. The Kolmogorov-Smirnov statistic was used for testing normality distributions in the data. Outlier detection was via the ROUT method set at Q = 1% (GraphPad Prism 8). Statistical differences between performance in the isolation and in the competition context were computed with independent samples Student’s *t* test for parametric testing and with the independent samples Mann-Whitney *U* test for non-parametric testing. Two-way ANOVA was used to study effects of incentives and sessions on success rate. Associations were quantified with Pearson’s correlation coefficient for normally distributed variable pairs and associations including not normally distributed variables were quantified with Spearman rank correlation coefficients. Correlation coefficients were compared according to Zou's confidence intervals. The relationships between metabolites and their ratios and the model parameters were tested with multiple linear regression with metabolite concentrations and social context (isolation vs. competition) as independent variables and model parameters as dependent variables, for normally distributed model parameters. Rank regression was used for model parameters that were not normally distributed. Due to lack of previously published data involving the variables measured in this study, all statistical tests were run two-sided and statistical testing was performed with an alpha level of 0.05.

**Supplementary References**

1. Spielberger CD. State-Trait anxiety inventory. Corsini Encycl Psychol. 2010:1–1.

2. Jackson DN. Personality research form manual. research psychologists press; 1974.

3. Houston J, Harris P, McIntire S, Francis D. Revising the competitiveness index using factor analysis. Psychol Rep. 2002;90:31–34.

4. Gilbert P, Price J, Allan S. Social comparison, social attractiveness and evolution: How might they be related? New Ideas Psychol. 1995;13:149–165.

5. Loy BD, O’Connor PJ. The effect of histamine on changes in mental energy and fatigue after a single bout of exercise. Physiol Behav. 2016;153:7–18.

6. Marques JP, Kober T, Krueger G, van der Zwaag W, Van de Moortele P-F, Gruetter R. MP2RAGE, a self bias-field corrected sequence for improved segmentation and T1-mapping at high field. Neuroimage. 2010;49:1271–1281.

7. Neto LL, Oliveira E, Correia F, Ferreira AG. The human nucleus accumbens: where is it? A stereotactic, anatomical and magnetic resonance imaging study. Neuromodulation Technol Neural Interface. 2008;11:13–22.

8. Gruetter R. Automatic, localized in vivo adjustment of all first-and second-order shim coils. Magn Reson Med. 1993;29:804–811.

9. Gruetter R, Tkáč I. Field mapping without reference scan using asymmetric echo-planar techniques. Magn Reson Med Off J Int Soc Magn Reson Med. 2000;43:319–323.

10. Xin L, Schaller B, Mlynarik V, Lu H, Gruetter R. Proton T1 relaxation times of metabolites in human occipital white and gray matter at 7 T. Magn Reson Med. 2013;69:931–936.

11. Govindaraju V, Young K, Maudsley AA. Proton NMR chemical shifts and coupling constants for brain metabolites. NMR Biomed. 2000:25.

12. Provencher SW. Estimation of metabolite concentrations from localized in vivo proton NMR spectra. Magn Reson Med. 1993;30:672–679.

13. Schaller B, Xin L, Gruetter R. Is the macromolecule signal tissue-specific in healthy human brain? A 1H MRS study at 7 tesla in the occipital lobe. Magn Reson Med. 2014;72:934–940.

14. Van Leemput K, Maes F, Vandermeulen D, Suetens P. Automated model-based bias field correction of MR images of the brain. IEEE Trans Med Imaging. 1999;18:885–896.

15. Provencher SW. LCModel & LCMgui user’s manual. LCModel Version. 2014:6–2.

16. Berchio C, Rodrigues J, Strasser A, Michel CM, Sandi C. Trait anxiety on effort allocation to monetary incentives: a behavioral and high-density EEG study. Transl Psychiatry. 2019;9.

2016;36:6623–6633.

**Supplementary Tables**

Supplementary Table 1 Personality questionnaires and anthropometric characteristics. Comparison between isolation (*N* = 15) and competition (*N* = 12) groups. There are no differences between the groups (Student’s *t* test, two-tailed). *M*, mean; *SD,* standard deviation.

|  | **Sample** | **M ± SD** | ***t* value** | ***p* value** |
| --- | --- | --- | --- | --- |
| State anxiety | Total | 30.00 ± 7.43 | -0.670 | 0.509 |
|  | Isolation | 29.13 ± 4.87 |  |  |
|  | Competition | 31.08 ± 9.90 |  |  |
| Trait anxiety | Total | 39.67 ± 8.04 | -0.047 | 0.963 |
|  | Isolation | 39.60 ± 5.87 |  |  |
|  | Competition | 39.75 ± 10.44 |  |  |
| Dominance motivation | Total | 9.52 ± 3.37 | -0.088 | 0.931 |
|  | Isolation | 9.47 ± 3.00 |  |  |
|  | Competition | 9.58 ± 3.92 |  |  |
| Competitiveness | Total | 46.70 ± 8.25 | -0.676 | 0.505 |
|  | Isolation | 45.73 ± 7.71 |  |  |
|  | Competition | 47.92 ± 9.07 |  |  |
| Self-perceived social rank | Total | 67.93 ± 8.11 | 0.193 | 0.849 |
|  | Isolation | 68.20 ± 7.93 |  |  |
|  | Competition | 67.58 ± 8.68 |  |  |
| Physical fatigue (state) | Total | 119.37 ± 67.55 | -0.489 | 0.629 |
|  | Isolation | 113.60 ± 70.96 |  |  |
|  | Competition | 126.58 ± 65.39 |  |  |
| Physical fatigue (trait) | Total | 3.44 ± 1.78 | 1.644 | 0.113 |
|  | Isolation | 3.93 ± 1.79 |  |  |
|  | Competition | 2.83 ± 1.64 |  |  |
| Physical activity | Total | 203.38 ± 37.33 | -0.641 | 0.528 |
|  | Isolation | 199.22 ± 23.01 |  |  |
|  | Competition | 208.59 ± 50.67 |  |  |
| Age (years) | Total | 23.44 ± 3.20 | 0.638 | 0.530 |
|  | Isolation | 23.80 ± 3.01 |  |  |
|  | Competition | 23.00 ± 3.52 |  |  |
| Height (cm) | Total | 177.76 ± 8.39 | 0.300 | 0.767 |
|  | Isolation | 178.20 ± 7.97 |  |  |
|  | Competition | 177.21 ± 9.22 |  |  |
| Weight (kg) | Total | 72.63 ± 6.75 | 0.090 | 0.929 |
|  | Isolation | 72.74 ± 7.41 |  |  |
|  | Competition | 72.50 ± 6.15 |  |  |
| Body mass index | Total | 23.07 ± 2.57 | -0.217 | 0.830 |
|  | Isolation | 22.98 ± 2.57 |  |  |
|  | Competition | 23.20 ± 2.67 |  |  |
| Maximal voluntary contraction (in kg) | Total | 25.52 ± 4.06 | -0.421 | 0.677 |
|  | Isolation | 25.22 ± 3.54 |  |  |
|  | Competition | 25.89 ± 4.77 |  |  |

Supplementary Table 2. Nucleus accumbens metabolite concentrations. Comparison between the isolation (*N* = 12) and competition group (*N* = 10). Absolute concentrations and CRLBs are given as mean (M) ± standard deviation (SD). The metabolite concentrations and their ratios did not differ between the groups (Student’s *t* test, two-tailed). Glu, glutamate; Gln, glutamine; GABA, gamma-aminobutyric acid; Metabolite concentrations stated in μmol/g. n.a.: not applicable.

| **Metabolite** | **Sample** | **M ± SD** | **CRLB (%) ± SD** | ***t* value** | ***p* value** |
| --- | --- | --- | --- | --- | --- |
| Glu | Total | 9.76 ± 1.14 | 2.36 ± 0.49 |  | |
|  | Isolation | 9.57 ± 1.04 | 2.42 ± 0.51 | -0.872 | 0.394 |
|  | Competition | 9.99 ± 1.27 | 2.30 ± 0.48 |  |  |
| Gln | Total | 4.34 ± 1.02 | 4.91 ± 0.75 |  | |
|  | Isolation | 4.25 ± 1.03 | 5.08 ± 0.67 | -0.443 | 0.663 |
|  | Competition | 4.45 ± 1.06 | 4.70 ± 0.82 |  |  |
| GABA | Total | 2.20 ± 0.52 | 11.32 ± 2.78 |  |  |
|  | Isolation | 2.18 ± 0.56 | 11.67 ± 2.99 | -0.202 | 0.842 |
|  | Competition | 2.22 ± 0.49 | 10.90 ± 2.60 |  |  |
| Gln/Glu | Total | 0.45 ± 0.07 | n.a. |  |  |
|  | Isolation | 0.44 ± 0.68 | n.a. | -0.080 | 0.937 |
|  | Competition | 0.44 ± 0.07 | n.a. |  |  |
| GABA/Gln | Total | 0.51 ± 0.11 | n.a. |  |  |
|  | Isolation | 0.52 ± 0.12 | n.a. | 0.096 | 0.924 |
|  | Competition | 0.51 ± 0.11 | n.a. |  |  |

Supplementary Table 3. Correlation matrix of accumbal Glu, Gln, and GABA with their ratios. Glu, glutamate; Gln, glutamine; GABA, gamma-aminobutyric acid. Pearson correlation coefficients are indicated. N = 27. Color coding corresponds to the Pearson correlation coefficient. **p* < 0.05; ***p* < 0.01; ****p* < 0.001; *****p* < 0.0001; uncorrected.

**Supplementary Table 4.** Means and principal component loadings for success rates of differently incentivized trials in different sessions. Principal component analysis (PCA) of 12 (4 sessions x 3 incentives) experimental performance measures. PCA was used to determine the effective dimensionality of the data considering twelve performance measures (see Methods), and which was reflected in the variance explained by each principal component (PC). We interpreted the component loadings as PC1 reflecting general performance success, PC2 capturing the incentive effect, and PC3-5 being ambiguous, thus, highlighting the limitations of PCA in producing clearly interpretable components.

|  | | | **Variance explained** | | | | |
| --- | --- | --- | --- | --- | --- | --- | --- |
| **12 performance measures** | | **Mean** | **PC1** | **PC2** | **PC3** | **PC4** | **PC5** |
|  |  |  | 46.8% | 13.7% | 11.2% | 7.3% | 6.1% |
| Session number and incentive size | S1, 0.2 | 0.607 | 0.2896 | 0.4816 | -0.0545 | -0.1467 | 0.1754 |
|  | S1, 0.5 | 0.830 | 0.2850 | 0.2695 | -0.0155 | 0.5558 | 0.1381 |
|  | S1, 1 | 0.911 | 0.2193 | -0.3227 | -0.2691 | -0.0262 | 06457 |
|  | S2, 0.2 | 0.533 | 0.3137 | 0.2387 | -0.3469 | -0.2425 | -0.1938 |
|  | S2, 0.5 | 0.711 | 0.3492 | 0.0563 | -0.0850 | -0.0668 | 0.2975 |
|  | S2, 1 | 0.837 | 0.1976 | -0.4440 | 0.0426 | -0.4664 | 0.0108 |
|  | S3, 0.2 | 0.667 | 0.3345 | 0.1663 | -0.0972 | -0.0982 | -0.3269 |
|  | S3, 0.5 | 0.882 | 0.2756 | -0.2610 | -0.2982 | 0.4226 | -0.4056 |
|  | S3, 1 | 0.889 | 0.3157 | -0.3971 | -0.1752 | 0.0100 | -0.1753 |
|  | S4, 0.2 | 0.526 | 0.3387 | 0.1690 | 0.3490 | -0.1613 | 0.1008 |
|  | S4, 0.5 | 0.711 | 0.2536 | -0.0514 | 0.5363 | -0.1745 | -0.2738 |
|  | S4, 1 | 0.800 | 0.2469 | -0.2222 | 0.5065 | 0.3786 | 0.1407 |

**Supplementary Table 5.** Spearman correlation coefficients between estimated parameter values, indicating high correlation only between the two stamina parameters.

| **ρ between** | **β** | **b** | **ε_spr_** | **ε_end_** |
| --- | --- | --- | --- | --- |
| **α** | 0.123 | 0.152 | 0.445 | 0.329 |
| **β** |  | 0.636 | 0.503 | 0.328 |
| **b** |  |  | 0.443 | 0.553 |
| **ε_spr_** |  |  |  | 0.792 |

**Supplementary Table 6.** Linear regression between estimated model parameters and measures of state and trait anxiety. For α, ε_spr_ and ε_end_ Spearman correlation was used and for β and b Pearson correlation.

| **Parameter\measure** | **Trait anxiety** | **State anxiety** |
| --- | --- | --- |
| α (utility curvature) | ρ = 0.091  p = 0.69 | ρ = 0.070  p = 0.76 |
| β (sigmoidal steepness) | r = -0.028  p = 0.90 | r = -0.14  p = 0.53 |
| b (effort cost baseline) | r = 0.014  p = 0.95 | r = -0.27  p = 0.22 |
| ε_spr_ (sprint stamina) | ρ = 0.21  p = 0.35 | ρ = -0.066  p = 0.77 |
| ε_end_ (endurance stamina) | ρ = 0.13  p = 0.55 | ρ = 0.012  p = 0.96 |

**Supplementary Table 7.** Multiple linear regression results with concentrations of different nucleus accumbens metabolites and metabolite ratios and task framing as independent variables and estimated model parameters as dependent variable. *P* values for each metabolite (or ratio) are shown on upper row in each cell followed by those of task framing in the lower row. For negative associations *p* values are shown in parentheses. Significant associations with metabolites (or ratios) are in italic. In addition to Gln/Glu results, we observed significant associations between ε_end_ and Gln as well as GABA/Gln.

| **Parameter\metabolite** | **Glu** | **Gln** | **GABA** | **Gln/Glu** | **GABA/Gln** |
| --- | --- | --- | --- | --- | --- |
| α (utility curvature) | (0.89)  (0.068) | 0.49  (0.050) | (0.68)  (0.058) | 0.13  (0.046) | (0.52)  (0.060) |
| β (sigmoidal steepness) | (0.31)  (0.15) | 0.86  (0.11) | (0.66)  (0.11) | 0.29  (0.096) | (0.56)  (0.10) |
| b (effort cost baseline) | (0.54)  (0.013) | 0.61  (0.009) | (0.33)  (0.009) | 0.26  (0.008) | (0.16)  (0.006) |
| ε_spr_ (sprint stamina) | 0.68  (0.080) | 0.094  (0.049) | 0.41  (0.085) | *0.010*  (0.045) | (0.27)  (0.085) |
| ε_end_ (endurance stamina) | 0.59  (0.35) | *0.040*  (0.25) | (0.89)  (0.40) | *0.003*  (0.29) | *(0.040)*  (0.38) |

**Supplementary Table 8.** Multiple linear regression results with occipital lobe Gln/Glu concentration ratios and experimental task framing as independent variables and estimated model parameter as dependent variable. For negative associations *p* values are shown in parentheses.

| **Model parameter** | **Association with Gln/Glu** | **Association with task framing** |
| --- | --- | --- |
| α (utility curvature) | (0.17) | (0.081) |
| β (sigmoidal steepness) | 0.11 | 0.27 |
| b (effort cost baseline) | 0.60 | (0.29) |
| ε_spr_ (sprint stamina) | (0.51) | (0.60) |
| ε_end_ (endurance stamina) | (0.85) | (0.91) |

**Supplementary Figures**

**Supplementary Figure 1.** **Voxel placement for** in vivo ^1^H-MR spectroscopy in the human male nucleus accumbens. MP2RAGE images with the volume of interest (14 x 10 x 13 mm^3^) in the left nucleus accumbens for localized ^1^H-MRS. The acquisition voxel is shown in red in all three planes, sagittal, axial, and coronal, from left to right.


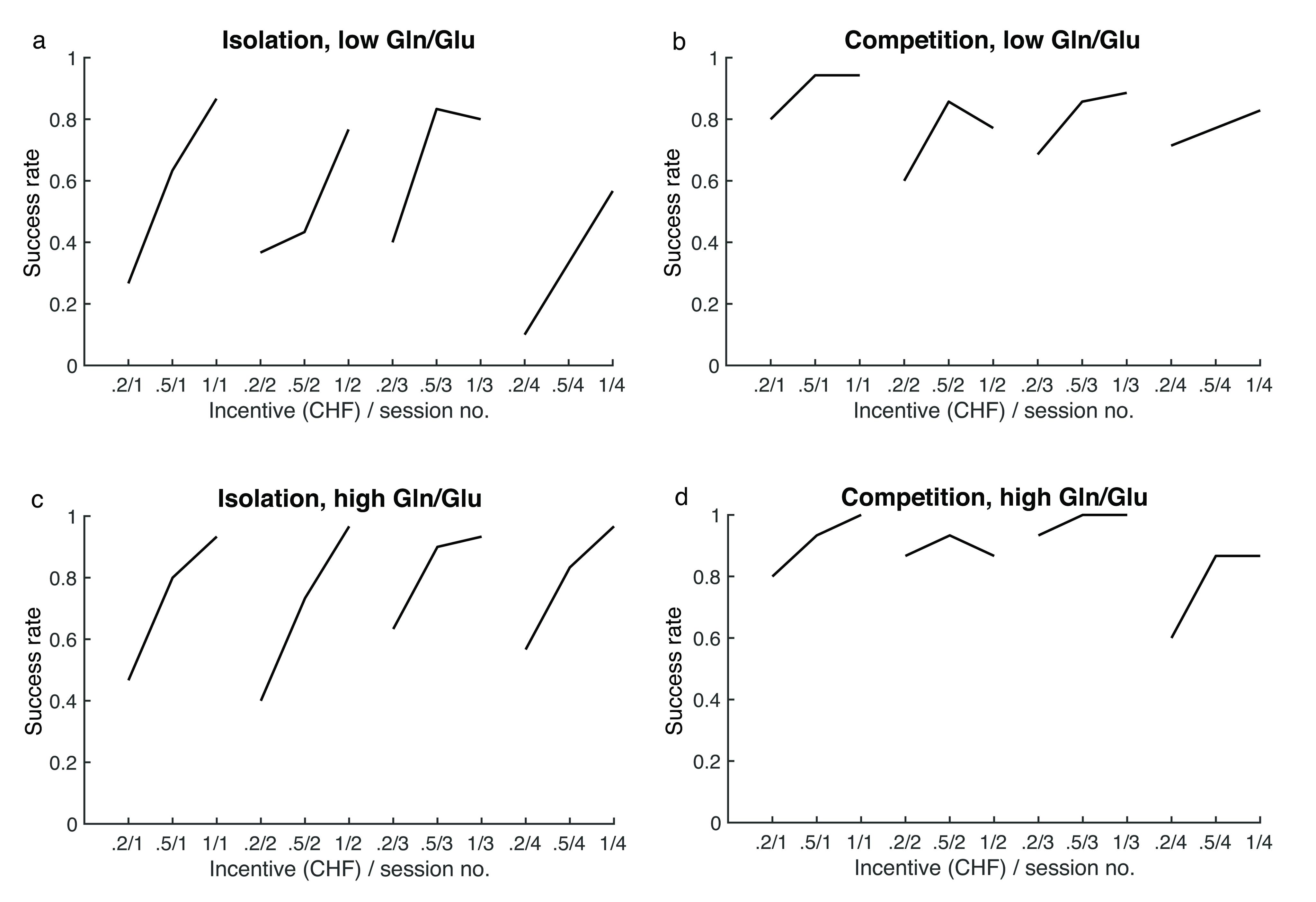


**Supplementary Figure 2.** Success rate in the isolation (a, c) and competition (b, d) context, for different incentives (0.2 CHF, 0.5 CHF, 1 CHF) and sessions (1-4), shown separately for participants with lower-than-average (a, b) and higher-than-average (c, d) accumbal Gln/Glu levels.


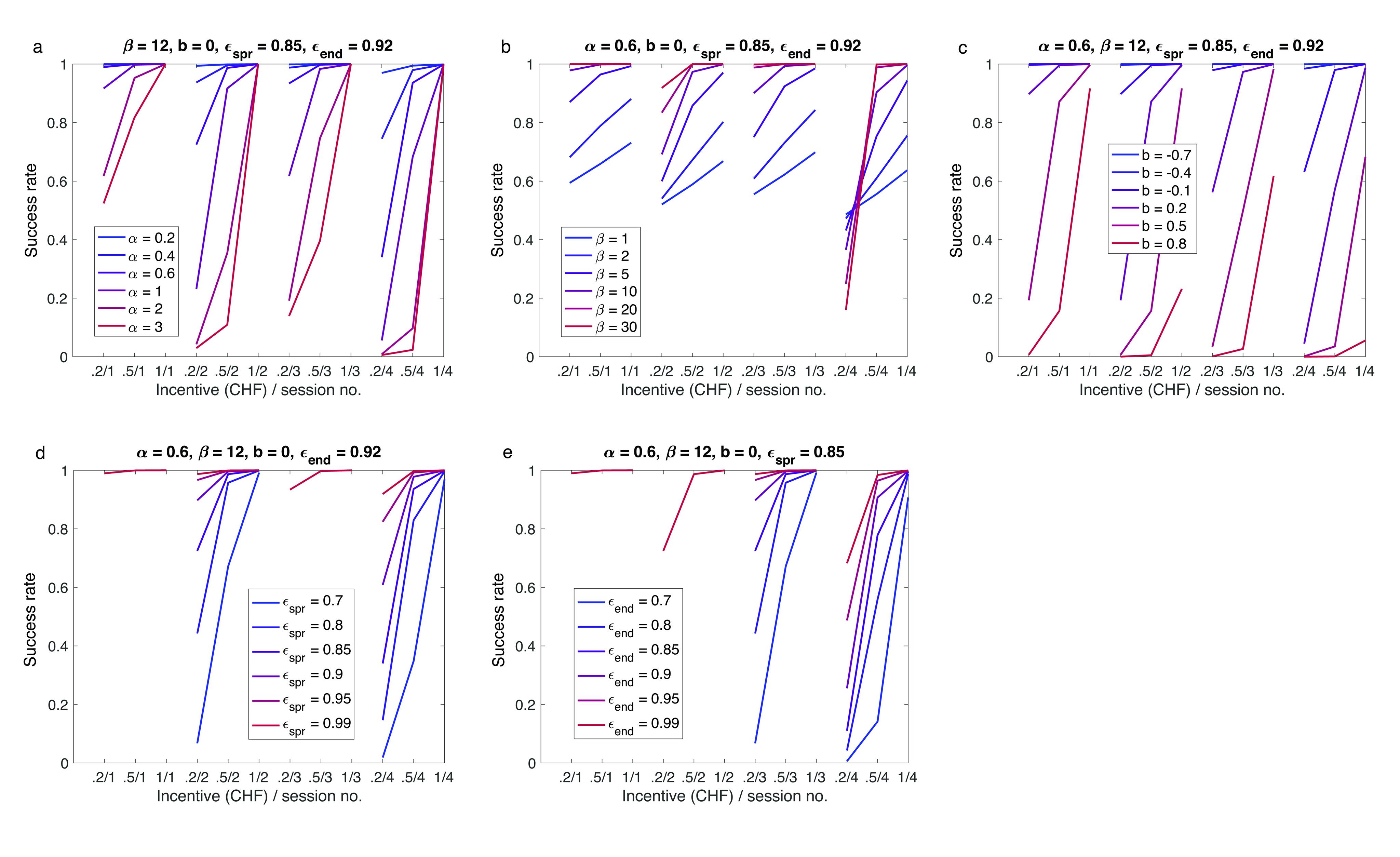


**Supplementary Figure 3.** Relationship between model parameters and success rate over sessions and for different incentives (0.2 CHF, 0.5 CHF and 1 CHF). Values of each parameter were varied within the most relevant range, whereas the values of the other parameters were fixed at a representative value. (a) Varying utility curvature only affected success rate of lower incentives with higher α corresponding to poorer performance. (b) Sigmoidal steepness controlled the contrast between low and high utilities, with high β values related to increased differences in success rate between them. (c) Effort cost baseline controlled overall performance, with low b values corresponding to high success rate and high b values to low. (d) Sprint stamina ε_spr_ determined the decline in performance between session 1 and 2 and between session 3 and 4 (i.e., with no break in between), with lower values representing a steeper decline. (e) Endurance stamina ε_end_ determined the decline in performance between the two experimental blocks (session 1 and 2, and session 3 and 4), separated by a 3 min break, with lower values representing a steeper decline. Although parameters β, b, ε_spr_ and ε_end_ differentially affect performance, this is not due to them differentially affecting utility, but because of the non-linearity introduced by the sigmoidal relationship between utility and performance.


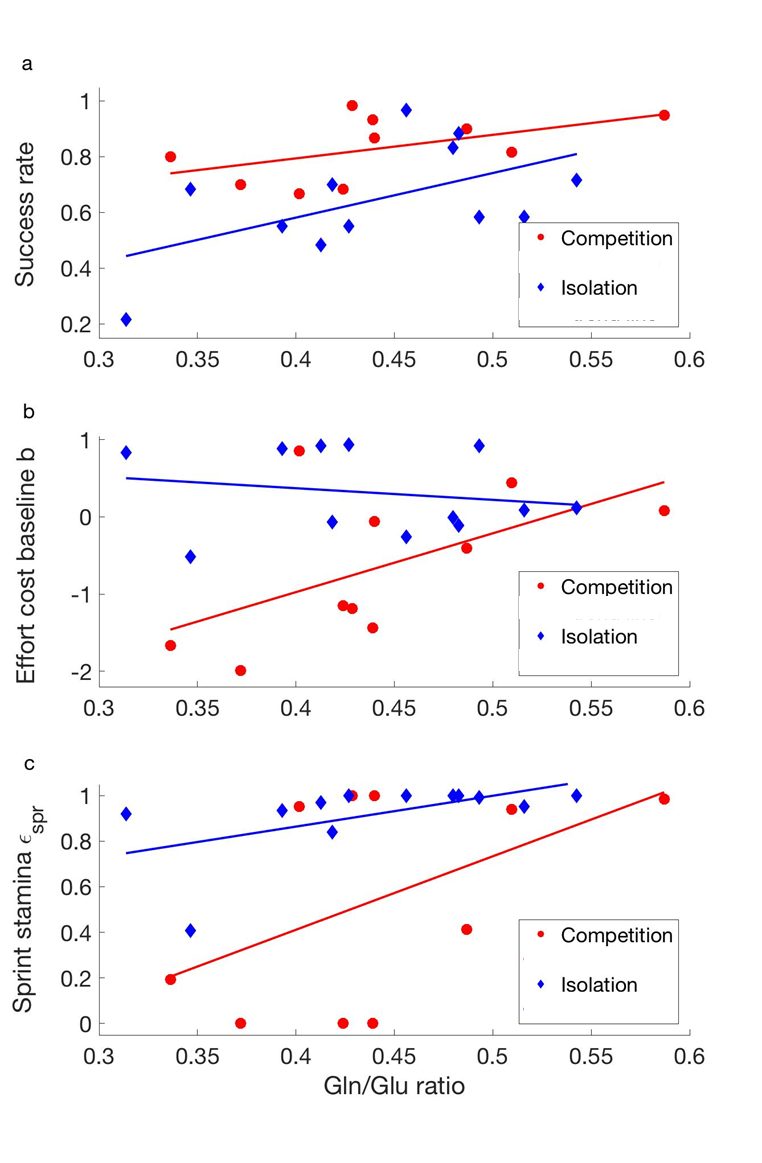


**Supplementary Figure 4.** Relationship between Gln/Glu ratio, social context and success rates as well as parameters b and ε_spr_. (a) For success rate there is a trend positive relationship with Gln/Glu ratio (p = 0.076). (b) For effort cost baseline b there is a negative relationship with the social context (p = 0.024) and a trend positive interaction between the social context and Gln/Glu ratio (p = 0.059). (c) For sprint stamina ε_spr_ there is a significant positive relationship with Gln/Glu ratio (p = 0.017).
